# Physiological Parameters of Sleep and the Risk of Obstructive Sleep Apnea in Competitive Athletes with Poor Sleep Quality

**DOI:** 10.3390/life15040610

**Published:** 2025-04-06

**Authors:** Feng-Yin Chen, Yung-An Tsou, Nai-Jen Chang, Wen-Dien Chang

**Affiliations:** 1 Department of Sport Performance, National Taiwan University of Sport, Taichung 404401, Taiwan; 71004002@gm.nuts.edu.tw; 2 Department of Otolaryngology-Head and Neck Surgery, China Medical University Hospital, Taichung 404328, Taiwan; d22052121@gmail.com; 3 Department of Sports Medicine, Kaohsiung Medical University, Kaohsiung 807378, Taiwan; njchang@kmu.edu.tw; 4 PhD Program in Biomedical Engineering, Kaohsiung Medical University, Kaohsiung 807378, Taiwan; 5 Department of Medical Research, Kaohsiung Medical University Hospital, Kaohsiung 807378, Taiwan; 6 Precision Sports Medicine and Health Promotion Center, Kaohsiung Medical University, Kaohsiung 807378, Taiwan

**Keywords:** apnea-hypopnea index, cardiopulmonary coupling, heart rate variability

## Abstract

This study aimed to explore the sleep conditions and obstructive sleep apnea (OSA) risk in athletes with poor sleep quality. Athletes with poor sleep quality before competition were recruited. Cardiopulmonary coupling analysis, the Pittsburgh Sleep Quality Index, Epworth Sleepiness Scale, and Insomnia Severity Index were used to assess and compare athletes at risk of OSA (apnea-hypopnea index (AHI) ≥ 5 events per hour) with those not at risk (AHI < 5 events per hour). Comparisons were made between the non-OSA group (n = 23) and the OSA risk group (n = 19, AHI = 10.79 ± 4.47 events per hour). The OSA risk group exhibited a significantly higher percentage of Stage 1 (S1) and Stage 2 (S2) sleep and greater heart rate variability (HRV) (*p* < 0.05). Positive correlations were found between AHI and the percentage of S1 and S2 sleep, low-frequency (LF), and the LF/HF ratio (*p* < 0.05). Conversely, significant negative correlations were observed between AHI and the percentage of Stage 3 (S3) and Stage 4 (S4) sleep, as well as HRV (*p* < 0.05). Athletes with poor sleep quality and high OSA risk demonstrated reduced parasympathetic activity, increased sympathetic activity, and affected sympathovagal balance during nocturnal HRV.

## 1. Introduction

When competitive athletes face high-intensity training or competitions and are under physical load and mental pressure, sleep disturbances or related sleep problems often occur. Sleep disturbances, including insomnia, day–night sleep cycle disorders, and daytime sleepiness, can lead to subjective fatigue, anxiety, or objective decline in physical efficiency and daily function [1]. A retrospective study found that elite athletes have a high proportion of sleep disturbances, with 16% to 70% suffering from insomnia [2]. These sleep disturbances had longer sleep latency and more sleep fragments, presenting the phenomenon of unstable sleep structure [2]. Sleep disturbances often lead to negative behaviors and related illnesses in young athletes and create negative health risks that affect social life or social conditions [3].

Regular and appropriate sleep is an important physiological repair mechanism for competitive athletes. A previous study also indicated that most athletes often reported no matter whether they had an adequate duration of sleep, they still had daytime sleepiness or poor spirits [4]. Obstructive sleep apnea (OSA) may be a potential reason to disturb sleep. Swinbourne et al. found that 38% of athletes reported snoring during sleep, and 8% of athletes felt that they suddenly stopped breathing and woke up during sleep [5]. Their study results found the incidence of OSA in athletes may be twice that of the general population [5]. The apnea-hypopnea index (AHI) is assessed as the average number of occurrences of apneas and hypopneas per hour in asleep and categorized into mild (5–15 events per hour), moderate (15–30 events per hour), and severe (above 30 events per hour) OSA risk [6]. It could be used to represent the severity of OSA risk. In the investigation on the risk of OSA in competitive athletes, referring to the survey on 22 professional rugby league players, it was found that 10 players had a mild or higher risk of OSA (AHI ≥ 5), the incidence rate was 45.45% [7]. However, there is still a lack of OSA risk screening and investigation for competitive athletes. The risk of OSA and the resulting sleep disturbances may affect physical recovery and sports performance and even affect long-term health in athletes.

Competitive athletes often experience poor sleep quality due to increased training volume or excessive pre-competition anxiety. Sleep is an important restorative mechanism for athletes, and it is thought to be beneficial to both physiology and psychology [8]. When athletes are faced with pre-competition stress, the results in insufficient sleep or poor sleep quality are often accompanied by. Leeder et al. used the Actigraph activity recorder to record the sleep conditions of 47 Olympians and compared them to a control group of 20 non-athletes. They found that elite athletes had poorer sleep quality and were less likely to fall asleep [9], and the causes of insufficient sleep or poor sleep quality in athletes often occurred before or during the competition [10]. Sometimes, athletes get excited before the competition due to anxiety and overtraining, resulting in poor sleep quality [10]. OSA risk is another factor affecting sleep quality, and this condition of abnormal breathing during sleep may tend to have abnormal physiological parameters of sleep. AHI is an index of the severity of OSA, and the effect of OSA on the physiological parameters of sleep is still unknown. OSA impacted sleep quality, as evidenced by various studies utilizing the Pittsburgh Sleep Quality Index (PSQI) and the Epworth Sleepiness Scale (ESS). A study by Mondal et al. involving 236 patients referred for polysomnography reported a mean PSQI score of 8.6 and a mean ESS score of 9.0, indicating poor sleep quality and excessive daytime sleepiness among individuals at high risk of sleep disorders [11]. A community-based study with 187 participants found that 50.8% of them had PSQI scores greater than 5, indicative of poor sleep quality, while 25.7% had ESS scores exceeding 10, reflecting significant daytime sleepiness [12]. To the best of our knowledge, there is limited investigation into the pre-competition sleep problems of competitive athletes and limited research on the impact of OSA risk in athletes. Limited research exists on pre-competition sleep disturbances and OSA risk among athletes, highlighting a critical gap in understanding their impact on health. Previous studies indicate that most athletes experience worsened sleep before important competitions [4], with 8% having witnessed apneic episodes [5]. Understanding the impact of OSA on athletes’ sleep is crucial for enhancing their well-being and competitive performance. So, the aim of our study was to explore sleep conditions and OSA risk in athletes with poor sleep quality before the competition. The physiological parameters of sleep were compared between the athletes with OSA and those at risk of non-OSA, and the relationship with AHI was explored.

## 2. Methods

### 2.1. Participants

Athletes with poor sleep quality before the competition were recruited athletes from the first-division sports terms of Taekwondo, wrestling, and track and field in the sports university. The study was approved by the Institutional Review Board, and each participant signed a written informed consent form. All of them were asked to fill in self-reported sleep quality, which was scored from 0 to 10 points, and respondent indicated how they rated their sleep quality during the past 7 days [13]. The inclusion criteria were that healthy athletes were continuously participating in competitive sports, represented self-reported sleep quality <5, and reported experiences of insomnia before the competition. Exclusion criteria were the absence of cardiovascular disease and the inability to complete this study. The cardiopulmonary coupling (CPC) analysis and subjective questionnaires for sleep quality assessments were collected and compared between non-OSA and OSA risk groups.

### 2.2. Study Procedures

**Figure 1:**
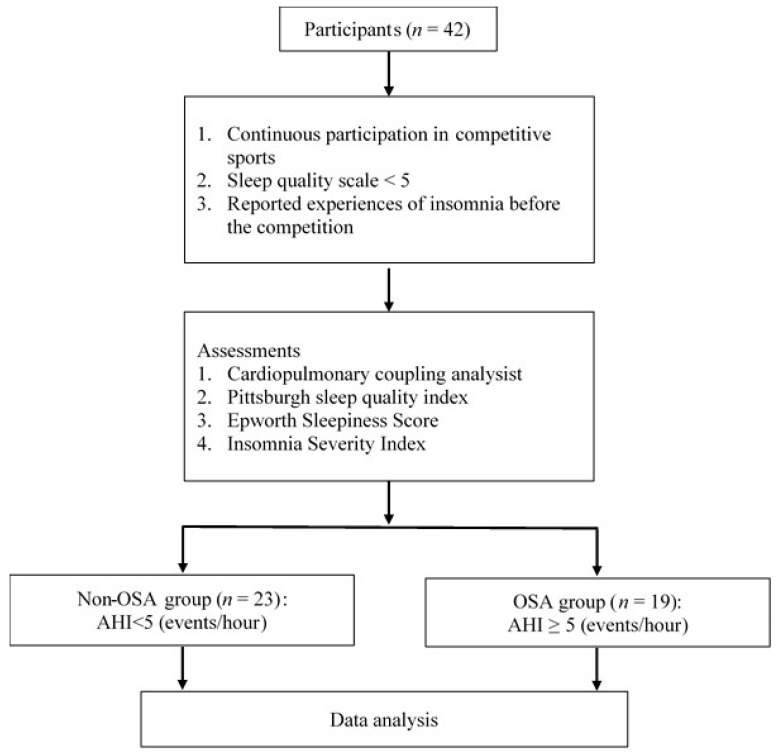
Subject testing process.

Before the study, demographic data in the body composition, height, weight, BMI, body fat, and muscle mass of all participants were measured and collected by filling in a questionnaire. They were asked to use a portable instrument of CPC (Largan Health AI-Tech Co., Taipei, Taiwan) to go home and sleep for one night at bedtime. Sleep data were collected by a home-based sleep quality monitoring device of CPC, which was used to collect electrocardiogram and physical activity data. The participants were asked to wear the CPC instrument during a one-night sleep at home. They were restricted from training, travel, diet, and caffeine intake during the test period. The physical data of sleep architecture, sleeping posture, and nocturnal heart rate variability (HRV) were collected by the instrument. The CPC analyzer generated values for the following bandwidths: 0.1–0.4 Hz, 0.01–0.1 Hz, and 0.0039–0.01 Hz, corresponding to high-frequency (HF) coupling and low-frequency (LF) coupling, which represent stable sleep, unstable sleep, and wake or rapid eye movement (REM) sleep, respectively. The AHI was recorded and calculated. The subjective questionnaires, i.e., PSQI, Epworth Sleepiness Score (ESS), and Insomnia Severity Index (ISI), were also used to assess the sleep quality (Figure 1). All assessments were conducted by an otolaryngologist in the university laboratory.

#### 2.2.1. Assessments

##### Cardiopulmonary Coupling Analysis

The CPC algorithm was used to measure the physical parameters during one night of sleep. The CPC was stuck below the third finger of the clavicle and between the sternum and the left mastoid in the participants. The electrocardiogram signals were collected and transferred to the cloud database. The measured data were analyzed for the nocturnal HRV and biophysical parameters in sleep by a specific algorithm. The HRV of CPC was classified as HF = 0.1–0.4 Hz and LF = 0.01 to 0.1 Hz by using the Fourier transform [14]. HF < 50% or LFC > 30% were suggestive of sleep instability [15]. The HRV, sleep architecture, sleeping posture, and AHI were calculated via the coherence of the coupling of the electrocardiogram and breathing signals. These CPC parameters were used to assess the severity of OSA risk and sleep stability.

##### Pittsburgh Sleep Quality Index

The PSQI is a standardized, self-administered questionnaire and is one of the most widely used sleep measures. Scores: subjective sleep quality, including sleep latency, sleep duration, habitual sleep efficiency, sleep disturbances, use of sleeping medication, and daytime dysfunction. Scores range from 0 to 3 and are summed to obtain a global score, which ranges from 0 to 21. Higher scores suggest greater sleep disturbance; a global score of more than 5 suggests a significant disturbance. The PSQI has high internal consistency reliability (Cronbach’s α = 0.83) and a fairly strong correlation coefficient (r = 0.85) for test-retest reliability [16].

##### Epworth Sleepiness Score

The ESS is a simple self-administered questionnaire. The subject was asked to rate, on a scale of 0–3, the chances that, in recent times, they would have dozed in eight specific situations. The total score range is from 0 to 24, with higher scores indicating greater sleepiness [17]. A total score of ESS ≥ 10 represents excessive sleepiness in the daytime. ESS has shown high internal reliability (Cronbach’s alpha = 0.88), supporting its use in sleep disorder assessments [18].

##### Insomnia Severity Index

The ISI is a simple assessment tool for assessing insomnia symptoms and daytime effects. Subjects completed the ISI, a seven-item tool that assesses an individual’s perception of insomnia. The first three items assessed insomnia symptoms in the early, middle, and late stages, and the higher the score, the higher the severity of insomnia. The last four items evaluated sleep satisfaction and dissatisfaction, the significance of sleep disturbance, sleep disturbance, and sleep disturbance to daily life, respectively. For these items, a Likert score (0 = not at all, 1 = a little, 2 = somewhat, 3 = much, 4 = very much) was used to assess the perception of insomnia. The ISI score range from scores 0 to 7 denotes “no insomnia”, scores of 8–14 denote “subthreshold insomnia”, scores of 5–21 denote “moderate insomnia”, and scores of 8–14 indicate “severe insomnia.” The ISI has good reliability and validity for the assessment of sleep problems and insomnia and has good internal consistency (Cronbach’s α = 0.74) [19].

### 2.3. OSA Risk Classification

The CPC-based analysis was used to classify the degrees of OSA risk. AHI was presented with the number of times of apnea during one night of sleep. AHI > 5 is widely used for diagnosing OSA [20]. The OSA risk group was defined as AHI ≥ 5 events per hour, and the non-OSA risk group was defined as AHI < 5 events per hour.

### 2.4. Statistical Analysis

SPSS version 25 (SPSS Inc., Chicago, IL, USA) was used for statistical analysis. The assessed data were analyzed by using descriptive statistics and represented as mean ± standard deviation (SD). Comparing the results between OSA risk and non-OSA groups, a t-test was used for the continuous variables, and the chi-square test was used for categorical variables. The effect size was calculated to measure the mean difference between the two groups. Spearman’s correlation test was used to analyze the relationship between physiological signal data and AHI. The correlation coefficient classification was performed as very high (r = 0.9~1), high (r = 0.7~0.9), moderate (r = 0.5~0.7), low (r = 0.3~0.5), and negligible (0~0.3) correlations, according to the study of Mukaka et al. [21]. α = 0.05 was considered statistically significant.

## 3. Results

Forty-two athletes with poor sleep quality participated in and completed the study. Twenty-three participants had non-OSA (AHI = 2.45 ± 1.59 events/hour), and 19 participants were in the OSA group (AHI = 10.79 ± 4.47 events/hour). We also noted that 45.23% (19/42) of athletes with poor sleep quality had mild OSA. In Table 1, there were no significant differences in demographic data between non-OSA and OSA risk groups (*p* > 0.05).

For sleep architecture, compared to the non-OSA risk group, participants in the OSA risk group had significantly longer duration in S1 and S2 and significantly less duration in S3 and S4 while asleep (Table 2, *p* < 0.05). We also found that the athletes with poor sleep quality in the OSA risk group had a significantly higher percentage of S1 and S2 and a lower percentage of S3 and S4 (*p* < 0.05). In Table 2, there was a significantly higher sleep duration in supine, and the sleeping posture in the S1 and S2 stages of sleep was noted in the OSA risk group (*p* < 0.05).

Compared with the non-OSA risk group, the higher LF and lower HF of HRV were found in the OSA risk group (*p* < 0.05, Table 3). The ratio of LF and HF in the OSA risk group was significantly higher than that in the non-OSA risk group (*p* < 0.05). However, there were no significant differences in subjective questionnaires, including PSQI, ISI, and ESS, between the two groups (*p* > 0.05). However, a total score of PSQI was calculated and categorized as indicating good sleep quality (a score of PSQI < 5) and poor sleep quality (a score of PSQI ≥ 5). The average scores of PSQI in the athletes with non-OSA and OSA risk were above 5, indicating they had poor sleep quality. The average scores of ESS > 10 were found and showed excessive daytime sleepiness. The average scores of ISI ranged from 5 to 21, which denotes moderate insomnia.

**Figure 2:**
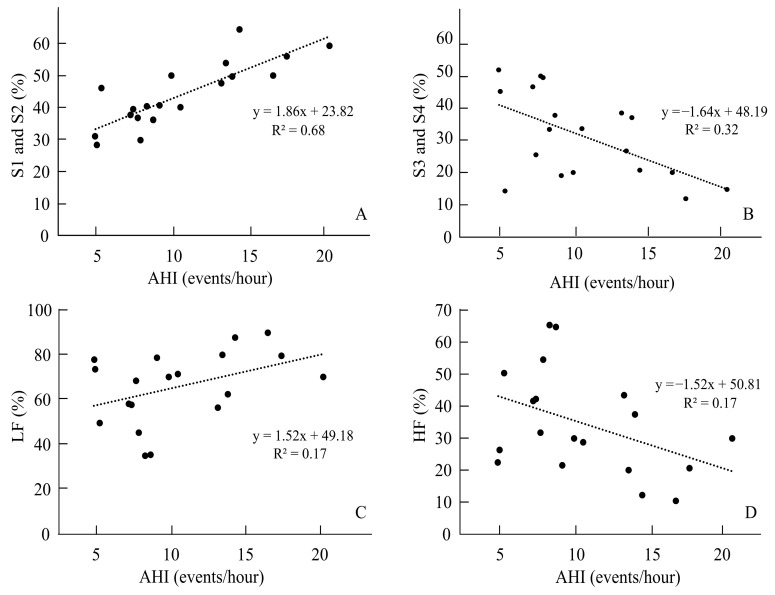
The relationship between AHI and sleep architecture (**A**,**B**) and HRV (**C**,**D**).

**Figure 3:**
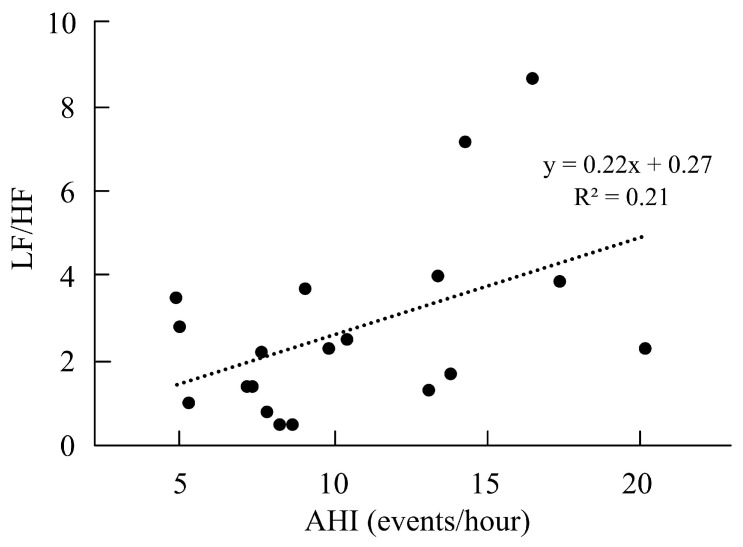
The relationship between AHI and LF/HF ratio.

Correlations between AHI and sleep architecture or HRV were analyzed in athletes with poor sleep quality and OSA (n = 19). The percentage of S1 and S2 of sleep architecture showed a positive correlation with AHI (r = 0.82, *p* = 0.001, Figure 2A), and the percentage of S3 and S4 showed a negative correlation with AHI (r = −0.57, *p* = 0.005, Figure 2B). However, there was no significant correlation between the percentage of REM and AHI (r = −0.13, *p* = 0.28). We also noted that LF and AHI had a positive correlation (r = 0.42, *p* = 0.03, Figure 2C), and HF and AHI had a negative correlation (r = −0.42, *p* = 0.03, Figure 2D). The ratio of LF and HF was also positively correlated with AHI (r = 0.46, *p* = 0.02, Figure 3). However, no significant correlations were observed between PSQI, ISI, or ESS and AHI (r = −0.12, *p* = 0.30; r = −0.24, *p* = 0.15; r = −0.001, *p* = 0.49, respectively).

## 4. Discussion

Our study investigated the pre-competition sleep problems and OSA risk in competitive athletes with poor sleep quality. We used CPC analysis and subjective questionnaires to survey the sleep architecture, HRV, and sleep quality. Comparing athletes with poor sleep quality in non-OSA (AHI < 5 events per hour) and OSA (AHI ≥ 5 events per hour) risk groups, differences in light sleep stage (S1 and S2), deep sleep stage (S3 and S4), and HRV were found between the groups. The changes in sleep architecture had moderate-to-high correlations with the severity of OSA risk.

Insomnia and OSA are sleep-related breathing conditions that affect sleep quality and fatigue recovery. It is also important for athletes because poor sleep and physiological recovery hinder their sports performance and health [22]. Previous studies reported that 15% of adults suffered from insomnia, and 50% of people worldwide were affected by OSA [23,24]. Some studies indicated that 27–37% of athletes had insomnia problems [25,26]. Caia et al. indicated that up to 45% of athletes suffered OSA, but only seven athletes were recruited for the survey [7]. In the current study, we surveyed 42 athletes and found that mild OSA risk was found in 45.23% of athletes with poor sleep quality. Montero et al. thought that the pressures from athletes themselves and other external criticism caused insomnia symptoms when the athletes focused on upcoming competitions [27]. The body position for sleeping had a relationship with the severity of OSA, and upper airway caliber and resistance often increased in a supine position for sleeping [28]. Avoiding a supine position while asleep is also a conservative treatment in patients with OSA [29]. In our findings, the athletes with poor sleep quality had a high OSA risk, but there was no significant difference in the duration in the supine position for sleeping between the non-OSA and mild OSA risk groups. However, we noted that the athletes with poor sleep quality and mild OSA had significantly greater duration and percentage of supine position in light sleep (S1 and S2 stages). The upper airway collapse occurs in light sleep and may affect the stages of change in the sleep cycle.

Although OSA was found mostly to be mild, even this degree of sleep-disordered breathing may cause a major disturbance in sleep, resulting in morning and daytime tiredness and also detrimental cardiorespiratory effects [30]. Like the general population, a bi-directional relationship between sleep and mental health has been observed among athletes [31]. However, they are more predisposed to sleep disorders. Insomnia symptoms are reported in 27% and 37% of athletes [32]. High stress, worry, and anxiety from competitive sports can increase sleep latency, further impairing sleep quality [33], while sleep maintenance insomnia has been reported in 77% of athletes [34]. However, small sample sizes, populations with specialized characteristics, and a lack of specific comparisons between persons with mild OSA and no OSA limit their interpretability.

CPC is a new computational analysis for measuring sleep architecture [35]. The CPC algorithm is stuck on the chest and is comfortable for recording the sleep architecture at home. This is the first study to use CPC for sleep measurement in athletes. We noted that the athletes with poor sleep quality and mild OSA had longer durations in light sleep (S1 and S2 stages). We also found a positive correlation with S1 and S2 of sleep architecture and AHI (r = 0.82) and a negative correlation with S3 and S4 of sleep architecture and AHI (r = −0.5). In the current study, AHI had a positive correlation with LF and LF/HF ratio but a negative correlation with HF among athletes with poor sleep quality and mild OSA. In the study of Park et al., they found that AHI had significantly positive correlations with LF (r = 0.39, *p* = 0.002) and LF/HF ratio (r = 0.61, *p* < 0.0001). These findings in athletes with mild OSA were the same as the results in the study of Park et al. [36]. Saul et al. indicated that partial closure of the upper airway caused hypoxemia and awakening for OSA to excite central chemoreceptors, resulting in a decrease in parasympathetic activity and an increase in sympathetic activity [37].

In our results on nocturnal HRV, there was no significant difference in SDNN from the time-domain analysis. However, there were significant differences in LF, HF, and LF/HF ratio from the frequency domain analysis between athletes with poor sleep quality combined with OSA and those without OSA. Roche et al. indicated that the standard deviation of NN intervals (SDNN) was a common HRV parameter and also a significant predictor for OSA [38]. Kim et al. also found that OSA patients had higher SDNN than healthy individuals [39]. However, we did not find a significant difference between OSA and non-OSA risk in the athletes. Because the athletes with mild OSA may not be severe OSA patients, the difference in SDNN was not found between the two groups in our study. For frequency domain analysis, LF and HF indicated sympathetic and parasympathetic activities, respectively. The ratio of LF and HF of HRV is meant for sympathovagal balance [40]. In the study of Aydin et al., the patients with severe OSA had higher LF and LF/HF ratios than those with mild OSA [41]. They also noted that LF and LF/HF ratios were increased in OSA patients compared with healthy individuals [41]. Narkiewicz et al. also supported that compared with healthy controls, LF and LF/HF ratio of HRV were higher in the patients with moderate-to-severe OSA [42]. Gula et al. found that the ratio of LF and HF of HRV in patients with mild OSA was higher than in healthy people [43]. Our study also noted the same findings: the athletes with poor sleep quality and mild OSA had significantly higher LF and LF/HF ratios and lower HF than the normal athletes. The athletes with poor sleep quality and mild OSA had higher parasympathetic nervous activity, lower sympathetic nerve activity, and poor sympathovagal balance in sleep. The parasympathetic nervous activity mediated heart rate, but sympathetic nervous activity released noradrenaline to increase heart rate and myocardium contracture velocity [44]. The ratio of LF and HF of HRV was higher to be calculated with the increase in LF and the decrease in HF. So, more sympathetic nervous activity and worse sympathovagal balance may affect falling asleep and into a deep sleep.

Sleep quality is a subjective experience and represents sleepiness, insomnia, and sleeplessness. Shahid et al. indicated that OSA patients had poor sleep quality, resulting in excessive daytime sleepiness, impacting their quality of life [45]. We used the PSQI, ESS, and ISI of subjective questionnaires to assess the sleep quality, sleepiness, and insomnia in the athletes with insomnia before the competition. Our findings show that all athletes experienced poor sleep quality, excessive daytime sleepiness, and moderate insomnia. However, there were no significant differences between those at risk of OSA and those not at risk. Additionally, the questionnaires showed no significant correlation with AHI, and self-reported sleep quality may not accurately represent physiological sleep disturbances. Miyahara et al. compared the patients with a low and high risk of OSA and revealed no significant differences in PSQI (*p* = 0.08) and ESS (*p* = 0.79) between the two groups [46]. Kim et al. also found that there were no differences in ISI among different severities of AHI in the OSA patients [47]. Some studies also indicated that a degree of AHI was not associated with the severity of subjective sleep quality and sleepiness [48,49]. These findings were similar to our results. The CPC analysis is an objective assessment in the current study and is thought to be useful in observing the sleep conditions in insomnia athletes.

However, there were some limitations of this study. First, the small sample size of our study may not be reflective of an athletic population. It is necessary to establish related data on OSA risk in larger samples of athletes. More risk factors of OSA should be collected and compared with OSA patients. It could be clearly understood that these factors influence sleep quality in athletes with insomnia before the competition. Second, it remains unclear whether poor sleep quality is due to insomnia, anxiety, training load, or other factors. Including a control group with good sleep quality can help strengthen the findings. Third, the method used may be less accurate than polysomnography for studying OSA and sleep problems, and only a single night of CPC analysis was completed in the current study. The multiple nights of assessments were suggested to decrease the intra-individual variability of one-single-night assessments.

## 5. Conclusions

The results of the current study revealed that 45.23% of athletes with poor sleep quality had OSA risk. The athletes with poor sleep quality and OSA risk represented more light sleep (S1 and S2) stages but fewer deep sleep (S3 and S4) stages during sleep. The degree of nocturnal HRV variability reflects its association with autonomic regulation. These changes in sleep architecture and HRV had correlations with AHI.

## Figures and Tables

**Table 1:** Demographics of the participants.

	Total (*n* = 42)	Non-OSA Risk (*n* = 23)	OSA Risk (*n* = 19)	*p*
Gender (male/female)	17/25	7/16	10/9	0.14
Age (years)	21.14 ± 2.25	20.74 ± 1.36	21.63 ± 2.97	0.24
Height (cm)	168.62 ± 7.83	167.41 ± 6.77	170.08 ± 8.92	0.29
Weight (kg)	60.90 ± 11.28	60.67 ± 9.61	61.18 ± 13.29	0.89
BMI (kg/m^2^)	21.36 ± 3.07	21.65 ± 2.82	21.01 ± 3.40	0.52
Body fat (%)	18.82 ± 8.78	20.91 ± 8.71	16.29 ± 8.41	0.09
Muscle mass (kg)	46.33 ± 8.46	44.84 ± 6.84	48.13 ± 9.98	0.23
Frequency of insomnia (times/week)	2.46 ± 1.35	2.59 ± 1.41	2.32 ± 1.29	0.52
Self-reported sleep quality	5.12 ± 1.47	5.22 ± 1.76	5.01 ± 1.05	0.62
AHI (events/hour)	6.23 ± 5.27	2.45 ± 1.59	10.79 ± 4.47	0.001 *

* *p* < 0.05.

**Table 2:** The results of sleep architecture and sleeping posture.

	Non-OSA Risk (*n* = 23)	OSA Risk (*n* = 19)	Cohen’s *d*	t	*p*
Sleep architecture					
Total time in bed (min)	438.26 ± 94.98	405.53 ± 86.51	0.36	1.17	0.25
Sleep period time (min)	418.96 ± 94.88	385.68 ± 88.63	0.36	1.17	0.25
Total sleep time (min)	393.74 ± 93.09	366.89 ± 86.32	0.29	0.97	0.34
Sleep efficiency (%)	89.70 ± 6.28	90.72 ± 4.82	0.18	−0.59	0.56
Duration in S1 and S2 (min)	109.83 ± 46.85	165.58 ± 48.56	1.16	−3.76	0.001 *
Percentage of S1 and S2 (%)	26.79 ± 10.11	43.98 ± 10.10	1.70	−5.49	0.001 *
Duration in S3 and S4 (min)	202.26 ± 68.09	116.05 ± 54.79	1.39	4.55	0.001 *
Percentage of S3 and S4 (%)	49.73 ± 11.74	30.41 ± 12.86	1.56	4.78	0.001 *
Duration in REM (min)	81.48 ± 34.91	85.26 ± 45.87	0.09	−0.03	0.77
Percentage of REM (%)	19.62 ± 6.42	21.66 ± 8.63	0.26	−0.85	0.40
Sleeping posture					
Duration in supine (min)	284.39 ± 107.36	274.47 ± 97.22	0.09	0.31	0.76
Supine in S1 and S2 (min)	72.78 ± 42.08	119.21 ± 49.06	1.01	−3.25	0.001 *
Supine in S3 and S4 (min)	149.00 ± 66.79	86.26 ± 46.41	1.09	3.58	0.001 *
Supine in REM (min)	52.96 ± 29.55	60.37 ± 42.27	0.20	−0.65	0.52
Duration in left side-lying (min)	50.83 ± 44.19	50.63 ± 36.93	0.01	0.02	0.99
Left side-lying in S1 and S2 (min)	16.78 ± 18.23	22.26 ± 19.94	0.28	−0.92	0.36
Left side-lying in S3 and S4 (min)	19.35 ± 25.52	12.47 ± 16.24	0.32	1.06	0.30
Left side-lying in REM (min)	12.00 ± 10.98	13.79 ± 13.39	0.14	−0.47	0.64
Duration in right side-lying (min)	64.17 ± 52.94	42.26 ± 38.13	0.47	1.56	0.13
Right side-lying in S1 and S2 (min)	15.96 ± 14.43	18.32 ± 20.03	0.13	−0.43	0.67
Right side-lying in S3 and S4 (min)	29.61 ± 30.72	12.42 ± 15.46	0.70	2.35	0.02 *
Right side-lying in REM (min)	13.96 ± 11.68	9.05 ± 8.07	0.48	1.60	0.12
Duration in prone (min)	9.70 ± 19.57	13.11 ± 30.41	0.13	−0.42	0.68
Prone in S1 and S2 (min)	3.17 ± 5.89	5.53 ± 16.56	0.18	−0.59	0.56
Prone in S3 and S4 (min)	3.09 ± 10.17	4.89 ± 11.59	0.16	−0.53	0.60
Prone in REM (min)	2.26 ± 4.72	1.95 ± 4.21	0.06	0.23	0.82

* *p* < 0.05. REM, rapid eye movement.

**Table 3:** The results of HRV and subjective questionnaires.

	Non-OSA Risk (*n* = 23)	OSA Risk (*n* = 19)	Cohen’s *d*	t	*p*
HRV					
Heart rate (beats/min)	60.57 ± 9.50	57.58 ± 9.17	0.32	1.03	0.31
SDNN (ms)	100.74 ± 34.88	97.00 ± 49.75	0.08	0.28	0.78
RMSSD (ms)	95.52 ± 44.63	74.89 ± 38.01	0.49	1.62	0.11
LF power (ms^2^)	2434.46 ± 3352.13	1959.81 ± 1914.66	0.17	0.57	0.57
LF (%)	53.82 ± 18.37	65.63 ± 16.09	0.68	−2.22	0.03 *
HF power (ms^2^)	1698.62 ± 1294.93	790.24 ± 595.24	0.90	3.00	0.01 *
HF (%)	46.18 ± 18.37	34.37 ± 16.09	0.68	2.22	0.03 *
LF/HF ratio	1.59 ± 1.25	2.72 ± 2.16	0.64	−2.01	0.04 *
pNN50 (%)	56.46 ± 15.54	48.98 ± 18.54	0.43	1.40	0.17
PSQI	9.61 ± 2.92	9.42 ± 2.81	0.06	0.21	0.83
ISI	13.70 ± 5.51	13.21 ± 3.74	0.10	0.33	0.74
ESS	11.17 ± 3.82	10.58 ± 2.55	0.18	0.60	0.55

* *p* < 0.05. SDNN, standard deviation of normal-to-normal intervals; RMSSD, root mean square of the successive differences; LF, low frequency; HF, high frequency; pNN50, proportion of NN50 divided by total number of normal-to-normal intervals.

## Data Availability

Data are contained within the article.

## Abbreviations

AHI	apnea-hypopnea index
CPC	cardiopulmonary coupling
HRV	heart rate variability
HF	high-frequency
LF	low-frequency
REM	rapid eye movement
PSQI	Pittsburgh Sleep Quality Index
ESS	Epworth Sleepiness Score
ISI	Insomnia Severity Index
SD	standard deviation
SDNN	standard deviation of NN intervals
